# A Method of Handling Alveolar Pyorrhea

**Published:** 1899-03

**Authors:** George T. Carpenter

**Affiliations:** Chicago


### A Method of Handling Alveolar Pyorrhea.

BY GEORGE T. CARPENTER, M.D., D.D.S., CHICAGO.

Presented to the Section on Stomatology at the Forty-ninth Annual Meeting of the American Medical Association, held at Denver, Colo., June 7-10, 1898.

In presenting this paper before this Section, I do so with a feeling that the practical side should not be lost sight of while we are eagerly searching out the scientific. Much has been written on the subject of pyorrhea alveolaris and there is a great difference of opinion still existing as to the etiology, pathology, diagnosis, and treatment of this disease. It is not my purpose in this paper to discuss the various points above mentioned, but to give a brief outline of a method of handling these cases which has given me the most satisfactory results. It has been my experience that different cases of pyorrhea differ in their treatment. One case may yield readily to a certain treatment, while another may be very persistent in its pathologic behavior. But thoroughness in diagnosis and treatment is the key to success. In diagnosing any case, get a history of it and decide on the predisposing cause, or cause outside the pocket, which is generally from local irritation caused by wooden toothpicks, ligature, wedging of teeth, or ill-fitting plates or crowns, accompanied with neglect of the mouth. These or other causes should be removed, and any constitutional predisposition should be corrected, and then we should secure the hearty co-operation of the patient. The disease once established is self-sustaining, and it is within the pocket that we must look for, find, and remove the exciting cause, which may be serumal deposits, pus-secreting membrane, foreign substances, or diseased or necrosed margins of process. To make a careful and thorough examination of the pockets I secure dryness with bibulous paper, protect the parts with a napkin, and coat the surrounding gum with cosmolin to protect the parts outside the pocket from the action of medicines. Now dry the pocket and pack with cotton slightly moistened with a 4 to 8 percent aqueous solution of cocain. After allowing this to remain several minutes, carefully remove the cotton. The pocket will be found to remain open so that an examination can be made with a mouth mirror. Deposits, if present, can be located before instruments are used for their removal. I operate on but one tooth at a time, or on the inter-proximal space between two teeth, doing all that I intend to do in that immediate locality before directing my attention to other teeth. All loose teeth having lost two-thirds or more of their natural support are extracted. The best instruments to use in the pockets are spoon-shaped excavators with the shanks bent so as to reach every part of the root or pocket. They must be kept sharp so that deposits can be easily removed and the root curetted. The soft parts and margin of process can also be curetted if necessary. The pocket should be cleansed with equal parts of peroxide of hydrogen and pasteurin, or 3 percent pyrozone and borolyptol, using a flat-pointed syringe, and pass to the bottom of the pocket. This syringe is designed especially to reach pyorrhea pockets, and can be used as successfully on posterior teeth as in the front part of the mouth, and fluids do not escape beside the point until the pocket is distended and all parts are reached. This will not only antiseptically cleanse, but will also mechanically carry out all free and loosened particles. The gums are now wiped dry and painted with tincture of iodin, which should be repeated about twice a week.* The tooth, if loose, is supported by bands or ligatures, and if moved by occlusion I grind the tooth or its antagonist sufficiently to prevent

*Dr. Talbot: Pyorrhea Alveolaris, International Dental Journal, April, 1896.

all movement, so as to procure rest for the affected parts. All subsequent treatment of the pocket should be made by the use of a thinly-shaved, hardwood toothpick, which can be moistened in the antiseptic and bent so as to reach any location. This should be passed into the pocket as deep as possible without injury to the tissue or causing pain or hemorrhage, and repeat the application once or twice a week, as long as the toothpick can gain entrance to the pocket. I find that it is seldom, if ever, necessary to make incisions through the gums into the pockets, and I but seldom resort to the use of dilute or stronger acids as an aid in softening or removing deposits or to establish resolution. Antiseptic and prophylactic conditions should be established and followed during treatment and indefinitely contained throughout life. I recommend a thorough rinsing of the mouth and the use of an antiseptic after each meal. I also recommend a medium stiff toothbrush to be carefully used once in twenty-four hours, and that at night before retiring. This time, once established, will not be substituted for other hours in the day. The brush will have time to dry, and the elasticity of bristles be restored, which will do the best work at the most needed time. A good dentifrice, with precipitated chalk as a base, should be used on the brush, after which one dram of pasteurin or borolyptol should be taken into the mouth and retained for from three to five minutes, at the same time moving the cheeks and lips so as to force it between the teeth and reach all parts of the mouth, then expectorate and retire without rinsing the mouth with water. The time spent in the successful treatment of this disease depends on the constitutional condition of the patient and the extent of the disease or the thoroughness in which the first operation is performed. The recurrence of the disease comes from a failure to remove the predisposing causes. The reproduction of gum tissue† in all inter-proximal spaces is essential to prevent the lodgment of food or injudicious use of the toothpick. I believe that a thorough removal of the cause, with antiseptic treatment and stimulation of the gum tissue, will cure the most persistent case of alveolar pyorrhea.

